# Mortality rates in children, young people, and young adults with JIA: an observational study using the Clinical Practice Research Datalink (CPRD)

**DOI:** 10.1016/j.ero.2025.04.001

**Published:** 2025-05-14

**Authors:** Lianne Kearsley-Fleet, Natasha Shaw, Jasmine Leslie, Michelle Johnson, Lucy R Wedderburn, Kimme L Hyrich, Jenny H Humphreys

**Affiliations:** 1 Centre for Epidemiology Versus Arthritis, Centre for Musculoskeletal Research, Division of Musculoskeletal and Dermatological Sciences, University of Manchester, Manchester Academic Health Sciences Centre, Manchester, UK; 2 Infection, Immunity and Inflammation Research and Teaching Department, University College London (UCL) Great Ormond Street Institute of Child Health, London, UK; 3 National Institute for Health and Care Research (NIHR) Biomedical Research Centre at Great Ormond Street Hospital, London, UK; 4 Centre for Adolescent Rheumatology Versus Arthritis at UCL, UCL Hospital and Great Ormond Street Hospital, London, UK; 5 NIHR Manchester Biomedical Research Centre, Manchester University Hospitals National Health Service (NHS) Trust, Manchester, UK; 6 Kellgren Centre for Rheumatology, Manchester Royal Infirmary, Manchester University Hospitals NHS Trust, Manchester, UK

## Abstract

**Objectives:**

Many rheumatological conditions have increased mortality rates; however, there is conflicting evidence regarding the impact of juvenile idiopathic arthritis (JIA) on mortality. This analysis aimed to calculate the all-cause mortality rate of patients with JIA, compared with (a) matched-control patients and (b) the general population mortality estimates.

**Methods:**

Using the UK General Practice data Clinical Practice Research Datalink, JIA cases starting <16 years old from England were included, matched 4-to-1 with non-JIA controls (birth year, gender, practice). Exposure started on first JIA-code date (matched-date for controls) or January 1, 2000, whichever was latest. Follow-up continued until December 31, 2018, or death, whichever was first. Cox-proportional hazards models compared mortality in JIA versus matched controls. JIA rates were stratified by systemic versus non-systemic JIA. Standardised mortality rates (SMRs) were generated for JIA versus general population estimates (calendar year, age, gender).

**Results:**

There were 4762 patients with JIA (30 deaths) and 13 957 matched controls (27 deaths); patient demographics were similar between cohorts. Mortality rate for JIA was 6.2/10 000 person years (95% CI: 4.3-8.9), and 1.9/10 000 person years (95% CI: 1.3-2.8) for matched controls; JIA patients had 3.3 times higher mortality (95% CI: 2.0-5.5). Patients with systemic JIA had 3.3 times higher mortality (95% CI: 1.6-6.9) versus those with non-systemic JIA. The SMR for JIA was 2.9 (95% CI: 2.1-4.2). Eighteen (60%) JIA deaths occurred before 2012.

**Conclusions:**

This analysis calculated mortality rates in young people with JIA in England. Death in young people with JIA is exceedingly rare. Higher rates were observed in patients with systemic JIA. Over half the deaths occurred prior to 2012 when biologic treatment, particularly for systemic JIA, was limited.

**WHAT IS ALREADY KNOWN ON THIS TOPIC**

• Juvenile idiopathic arthritis (JIA) is an autoimmune condition that starts in childhood; diagnosed before the age of 16 years. It affects approximately 1 to 2 in 2000 children and young people.
• Recent research using healthcare data from Nordic countries found conflicting results of whether those with JIA have increased mortality versus the general population.

**WHAT THIS STUDY ADDS**

• This research used the Clinical Practice Research Datalink (CPRD) national database of routinely collected data from primary care practitioners for approximately 7% to 13% of the UK population.
• JIA patients had 3 times higher mortality rates compared with the matched-control patients, and with the general population.
• Patients with systemic JIA had 3 times higher mortality versus those with non-systemic JIA, and 3 in 5 of the JIA deaths occurred before 2012.

**HOW THIS STUDY MIGHT AFFECT RESEARCH, PRACTICE OR POLICY**

• Death in young people with JIA is exceedingly rare, with slightly higher rates observed in patients with systemic JIA.
• Over half the deaths occurred prior to 2012 when biologic treatment, particularly for systemic JIA, was limited.

Alt-text: Unlabelled box

## INTRODUCTION

Juvenile idiopathic arthritis (JIA) is an autoimmune condition that starts in childhood; diagnosed before the age of 16 years. It affects approximately 1 to 2 in 2000 children and young people [1]. It is a heterogeneous condition consisting of 7 International League of Associations for Rheumatology (ILAR) categories with varying disease characteristics, including but not limited to arthritis, fever, rash, enthesitis, psoriasis [2].

Rheumatological conditions are known to impact mortality. Evidence in adults with rheumatoid arthritis using primary care data suggest an all-cause mortality rate of 2.7 per 10 000 person years (95% CI: 2.6-2.8), with rheumatoid arthritis being associated with a 15% excess risk of mortality after 2006 compared with age, gender, and practice matched-control patients [3].

A recently published study of nationwide healthcare data from Denmark looked at multiple immune-mediated inflammatory diseases with a median of 11 years of follow-up per patient [4]. This cohort included 5744 children and young people with JIA (79% diagnosed between 2000 and 2017), of which 61% were female, and the median age at diagnosis was 9.6 years. The all-cause mortality rate for those with JIA was 4.4 per 10 000 person years (95% CIs unavailable). The authors identified 2.3 additional deaths per 10 000 patient years in those with JIA compared with the reference population (adjusted for age, sex, decade of diagnosis, and family income); adjusted hazard ratio was 2.3 (95% CI: 1.6-3.4). In contrast, a similar nationwide healthcare data study in Finland, which included 4180 children and young people with JIA (diagnosed between 2000 and 2014) with an average follow-up of 6.6 years per patient (total person years 28 941) [5], found that ‘the relative risk of death in the JIA group was not elevated; hazard ratio was 1.44 (95% CI: 0.70-2.95)’. However, neither of these 2 studies reported disease severity or ILAR subtype data for these patients; therefore it was unknown whether these patients had severe disease or more severe subtype systemic JIA.

The aim of this analysis was to calculate the all-cause mortality rate of children, young people, and young adults with JIA in the England compared with (i) matched-control patients without JIA and (ii) the general population based on age and gender.

## METHODS

### Data sources

This analysis used data from primary care electronic patient records in the UK; the Clinical Practice Research Datalink (CPRD). The CPRD comprises 2 datasets of routinely collected clinical data from primary care practitioners caring for approximately 7% to 13% of the UK population [6,7]; CPRD Aurum, which derives information from practices using the EMIS software, and CPRD GOLD, which derives information from practices that use the Vision software. In this analysis, the datasets were combined into a single dataset. For patients who could be identified in both GOLD and Aurum (ie, either the patient moved practices, or the practice switched software), only the data from Aurum were used. Linkage is available for the CPRD to the NHS England Hospital Episode Statistics (HES) data for inpatient admissions and outpatient clinics at English hospitals. This analysis included data from the linked data only, thus only patients from England were included. The CPRD has pre-existing ethical approval from a National Research Ethics Service Committee, which covers all observational research using anonymised CPRD data such as this study. The study was approved by the CPRD Independent Scientific Advisory Committee (protocol number 19_060).

### Patient inclusion

Patients with JIA were identified in both Aurum and GOLD datasets using a predefined Read code list for JIA; a broad list aiming to encompass all mention of arthritis that could be diagnosed in childhood (see Supplementary Table S1 and S2). All patients identified as having arthritis were then validated, as previously published [1], by 1 of 2 criteria using the NHS HES-linked data: (1) they had visited a rheumatologist and/or ophthalmologist 3 times within 12 months or (2) they had a hospital admission with a documented ICD-10 code for juvenile arthritis (ICD-10 heading of M08 (Juvenile arthritis) or M09). Although validation could only occur during the period of HES data availability (2003 until 2017), all available CPRD time was included to capture the whole JIA journey. For patients with JIA, both prevalent and incident cases were included. Non-JIA control patients were matched 4-to-1 based on year of birth, gender, and practice. Control patients had to have observations between 2000 and 2018. Any control patients who were identified to have JIA were excluded from the control cohort. Control patients could be included twice if they matched with 2 JIA patients, with matched JIA onset date being different between the 2 matched pairs. All patients were from England due to the requirement for linkage with NHS hospital data.

### Outcome and exposure

The outcome for this analysis was death, collected either in the CPRD dataset or through the NHS-linked data. If a different date was recorded in the CPRD and the NHS datasets, the earliest date of death was chosen for analyses. Cause of death was obtained from the NHS-linked death data (ICD-10 codes). For all patients, exposure started on the date of the first JIA code (or matched date for control patients) or January 1, 2000, whichever was latest. All patients were observed until the CPRD data cut-off date (December 31, 2018) or death, whichever was first.

To provide data on mortality rates in the general population, the Office for National Statistics (ONS) mortality rates for England and Wales were used [8] (note England only rates were not available). Mortality rates were identified for the years 2006 until 2014, presented as mortality rates per 1000 person years for each calendar year, by year of age and gender (male or female). For the years 2015 through to 2018, ONS provided the number of deaths and population estimates for each calendar year, year of age, and gender [9]. Mortality rates were then manually generated and transformed to rates per 1000 person years. For years 2000 to 2005, the 2006 mortality rates were used as ONS specific mortality rates for these years were unavailable.

### Statistical analysis

Patient characteristics of the children and young people with JIA and non-JIA controls were described as numbers (percentages) for categorical data and median (IQR) for continuous data. Crude mortality rates were calculated for both cohorts. Kaplan–Meier survival plots were generated for JIA and non-JIA control patients. Cox-proportional hazards model was used to compare mortality in patients with JIA compared with the non-JIA control patients. Standardised mortality ratios (SMRs) were calculated for both cohorts, comparing mortality rates with the general population estimates.

In addition, JIA patients were stratified by whether they had systemic JIA or not. Patients with a systemic JIA code were identified via 2 methods. First was using the ‘CPRD unique code for the medical term selected by the general practitioner from the predefined JIA code list. Read-codes with either ‘still’ or ‘systemic’ in the descriptor were highlighted as patients with systemic JIA. These included ‘Adult still’s disease’, ‘Adult-onset still’s disease’, and ‘Juvenile rheumatoid arthritis—still’s disease’ [*N* = 450]. Second, using the HES outpatient diagnoses (up to 12 diagnoses can be recorded per visit) or admitted patient care ICD-code, for either ‘Juvenile arthritis with systemic onset (M08.2)’ or ‘Adult-onset Still disease (M06.1)’ [*N* = 215; 147 additional to the above, 5 of which were patients with adult-onset Still's disease]. However, all patients had to have first met the patient inclusion criteria as above. Crude mortality rates were generated and hazard ratios used to compare mortality rates in those with systemic JIA versus those without. They were not compared with the control cohort or the general population.

#### Covariates

Date of birth was calculated using the month and year of birth provided. The 15th of the month was imputed as the day of birth for all patients. Patients with a missing month were imputed as June. For the survival analysis, if the start of follow-up (ie, CPRD entry date) was before the date of birth due to the assumptions above (by a maximum of 6 months as per the imputation assumptions), the date of birth was updated to match start of follow-up.

Ethnicity was obtained from CPRD observation read codes, and the ethnicity variable for HES outpatient and admitted patient care records. Values were classified using the ONS England ethnic group classification [10] of 5 first-tier categories: White, Black, Asian, Mixed, other, or missing. If there were conflicting records, the most frequently recorded ethnicity was used as per the CPRD guidelines [11]. Where there was not a most frequently recorded ethnicity, the CPRD record was used preferentially over the HES record, except if ‘other’ was recorded in CPRD, in which case the HES record was used. The CPRD Ethnicity Record sources underlying data from HES Copyright © 2024], re-used with the permission of The Health & Social Care Information Centre. All rights reserved.

From NHS-linked data, Index of Multiple Deprivation (IMD) was obtained for each patient. These were categorised into quintiles. In addition, 2011 Rural-Urban Classification at LSOA level was identified for each GP practice.

Asthma was included as a comorbidity to observe if the cohorts are comparable with respect to comorbidities. Patients with ≥1 Read code for asthma (see Supplementary Table S3 and S4) prior to the age 18 were identified as having childhood asthma.

#### Sensitivity analysis

To investigate the impact of the changing landscape of treatments for JIA, particularly an expansion of biologic therapy options for systemic JIA, as part of a sensitivity analysis the analysis was divided into time categories: ‘2000 until 2011’ versus ‘2012 until 2018’, based on the date that tocilizumab was European Medicines Authority (EMA) approved for systemic JIA patients [12] (19 May 2011) and allowing for approval time within NHS trusts. Following the initial ‘setting’ of the survival data in the main analysis, exposure was divided into the 2 categories. For ‘2000 until 2011’, exposure started on the date of the first JIA code (or matched date for control patients) or January 1, 2000, whichever was the latest. Patients were observed until December 31, 2011, or death, whichever was first. For ‘2012 until 2018’, exposure started on the date of the first JIA code (or matched date for control patients) or January 1, 2012, whichever was latest. Patients were observed until December 31, 2018, or death, whichever was first. Patients could contribute to both time periods if they had exposure time relevant to each. Crude mortality rates were calculated for both time periods. Cox-proportional hazards model was used to compare mortality in patients with JIA between 2000 and 2011 compared with 2012 until 2018. SMRs were calculated for both time periods, comparing mortality rates with the general population estimates.

### Patient and public involvement statement

This work has been supported by 3 patient partners, all co-authors on the manuscript.

## RESULTS

### Patient characteristics

A total of 4762 children and young people with JIA were identified (Table 1); 58% female, 86% were of White ethnicity, median age at first JIA code was 7 years (IQR: 3-11), and median age at the end of follow-up (December 31, 2018, or death) was 18 years (IQR: 13-24). There were 597 (13%) patients with a systemic JIA code. A total of 13 957 matched controls were included; 58% female, 87% were of White ethnicity, median age at matched date for JIA case was 9 (IQR: 5-12), and median age at last follow-up (December 31, 2018, or death) was 18 years (IQR: 13-24). National quintiles of IMD in both JIA and the matched-control cohort were distributed similarly to the general population, and 87% attended GPs in urban areas. Proportion of people in each ethnic group was similar to the 2011 ONS England and Wales census figures [13].

**Table 1:** Patient characteristics of JIA matched-control patients, including all patients, and those who died

	Patients with JIA		Control patients	
	All patients	Died	All patients	Died
Number of patients	4762	30	13 957	27
Age at first JIA code (JIA) or matched-date (controls) (years)	7 (3-11)	8 (5-12)	9 (5-12)	13 (9-14)
Age at last follow-up (years)	18 (13-24)	15 (11-20)	18 (13-24)	20 (18-23)
Available follow-up (years)	9.9 (5.1-15.2)	-	10.1 (5.2-15.3)	-
Gender				
Male	2003 (42%)	12 (40%)	5880 (42%)	13 (48%)
Female	2759 (58%)	18 (60%)	8077 (58%)	14 (52%)
Systemic JIA code				
Yes	597 (13%)	11 (37%)	-	-
No	4165 (87%)	19 (63%)	-	-
Ethnicity	*N* = 4648	*N* = 30	*N* = 12 112	*N* = 25
White	4014 (86%)	22 (73%)	10 494 (87%)	23 (92%)
Black	170 (4%)	<5	455 (4%)	<5
Asian	323 (7%)	<5	784 (6%)	<5
Mixed	124 (3%)	<5	284 (2%)	<5
Other	17 (<1%)	<5	95 (<1%)	<5
Index of Multiple Deprivation Quintile	*N* = 4758	*N* = 30	*N* = 13 946	*N* = 27
Q1 (least deprived)	1028 (22%)	5 (17%)	3169 (23%)	<5
Q2	927 (19%)	5 (17%)	2659 (19%)	5 (19%)
Q3	901 (19%)	8 (27%)	2628 (19%)	6 (22%)
Q4	922 (19%)	6 (20%)	2667 (19%)	<5
Q5 (most deprived)	980 (21%)	6 (20%)	2823 (20%)	11 (41%)
Childhood asthma				
Yes	189 (4%)	-	731 (5%)	-
No	4573 (96%)	-	13 226 (95%)	-

JIA, juvenile idiopathic arthritis.

Results expressed as number (percentage) or median (interquartile range). Numbers less than 5 have been suppressed to protect patient confidentiality; displayed as ‘<5’.

### Mortality

For the 4762 patients with JIA, over 48 234 years of follow-up (median: 9.9 years per patient), there were 30 deaths (Table 2). Mortality rate was 6.2 per 10 000 person years (95% CI: 4.3-8.9). For the 13 957 control patients, over 142 773 years of follow-up (median: 10.1 years per patient), there were 27 deaths. Mortality rate was 1.9 per 10 000 person years (95% CI: 1.3-2.8). JIA patients had a 3.3 times higher mortality rate than the control patients (95% CI: 2.0-5.5). Patients who died in each cohort were similar to the whole cohort with respect to gender, IMD, and area of practice. Of the JIA patients, more patients who died had a systemic JIA code (30%) versus the proportion in the whole JIA cohort (9%). In addition, control patients who died were older (median age: 20 years old) versus the JIA cohort (median age: 15 years).

**Table 2:** Mortality rates for JIA and match-control patients

	Patients with JIA	Control patients
Number of patients	4762	13 957
Total follow-up available (years)	48 234	142 773
Median (IQR) follow-up per person (years)	9.9 (5.1-15.2)	10.1 (5.2-15.3)
Number of deaths	30	27
Rate (95% CI) per 10 000 years	6.2 (4.3-8.9)	1.9 (1.3-2.8)
Hazard ratio (95% CI)	3.2 (2.0-5.5)	[reference]
SMR	2.9 (2.1-4.2)	0.9 (0.6-1.3)

CI, confidence intervals; IQR, inter-quartile range; JIA, juvenile idiopathic arthritis; SMR, standardised mortality rate.

The SMR of JIA compared with the general population (based on age, gender, and calendar year) was 2.9 (95% CI: 2.1-4.2), ie, those with JIA had 2.9 times higher mortality rates versus what you would expect given their age, gender, and calendar year. No difference in mortality rates of the control patients was found versus the general population; SMR 0.9 (95% CI: 0.6-1.3).

Stratified by systemic JIA, mortality rates were higher in those patients with a systemic JIA code (14.6 per 10 000 person years) compared with non-systemic JIA patients (4.7 per 10 000 person years); hazard ratio 3.3 (95% CI: 1.6-6.9). In addition, both SMRs were raised; systemic JIA SMR 6.6 (95% CI: 3.7-11.9), non-systemic JIA SMR 2.2 (95% CI: 1.4-3.5).

For the 30 JIA patients who died over the observation period, 18 (60%) deaths occurred before 2012 (9 of which were systemic JIA patients). Cause of death was known in 29 of the patients, of which 7 were from developmental, neurological, or congenital conditions; 5 were from infections; and the remainder comprised malignancies, pulmonary disease, cardiovascular, accidental, and allergy at frequencies of less than 5 cases per cause. Causes of deaths were broadly similar between the JIA and the control cohorts.

### Sensitivity analysis

Stratified by time-period, mortality rates were lower in those JIA patients who continued follow-up time between 2012 and 2018 (4.4 per 10 000 person years; IQR: 2.5-7.7) compared with those followed between 2000 and 2011 (8.7 per 10 000 person years; IQR: 5.5-13.8), although CIs overlapped (*P* = .06). Both SMRs were raised; between 2000 and 2011, SMR was 4.5 (95% CI: 2.8-7.1), whereas between 2012 and 2018, SMR was 2.0 (95% CI: 1.1-3.4).

## DISCUSSION

This is the first analysis of mortality rates in children, young people and young adults with JIA in a primary care population of England. The all-cause mortality rate for all patients was 6.2 per 10 000 person years, with those with systemic JIA having a higher mortality rate (14.6 per 10 000) than non-systemic JIA patients (4.7 per 10 000).

All-cause mortality rate for JIA in this analysis was higher compared with a recent publication from Denmark using national registry data (4.4 per 10 000) [1]. Inclusion into both studies was similar, with median age at diagnosis 7 years in the current analysis, and 9.6 years in the Danish study. Both followed patients over time into adulthood, with the current analysis having a median of 9.9 years, and the Danish study having 11 years of follow-up; however, the Danish follow-up number represented all the patients with paediatric-onset immune-mediated inflammatory disease of which only 50% were JIA (23% Crohn’s disease, 22% ulcerative colitis) so the exact figure for follow-up in the JIA patients specifically was unknown. In addition, the current analysis mortality rates were lower than another UK estimate based only on patients exposed to disease-modifying anti-rheumatic drug therapy (both methotrexate or biologic therapy) which was 11 per 10 000 person years [14]. When the current analysis was stratified by time-period, JIA patients appeared to have slightly lower mortality rates in more recent years (between 2012 and 2018) perhaps coinciding with the availability of JIA treatment more targeted for systemic JIA, compared with mortality rates between 2000 and 2011.

The crude mortality rate was 3.3 times higher than the matched non-JIA control cohort, and similarly 2.9 times higher compared with the general population base on what you would expect given their age and gender. A previous publication analysing mortality in JIA patients from the UK exposed to disease-modifying anti-rheumatic drug therapy also found a raised SMR [14], which was particularly high among those patients with systemic JIA. In the current analysis, a similar difference was seen between systemic and non-systemic JIA patients; systemic JIA patients had an SMR of 6.6, whilst non-systemic JIA patients had an SMR of 2.2. Compared with the control cohort, there were approximately 4 additional deaths per 10 000 person years; one additional death for every 2500 years of follow-up. These estimates are double of what was reported in Denmark [4].

This analysis was completed in a large national dataset, largely representative of UK general practice data. It used a validated code list to identify JIA patients, and a matched non-JIA control cohort for comparisons. However, it is possible that this analysis has missed some of the milder cases of JIA where only a few joints were affected for a short amount of time, which might not have triggered a referral to a rheumatologist, or where the child did not require ongoing rheumatology care. Thus, the population studied is likely to represent more severe JIA in the population. There was missing data for ethnicity for 2% of the JIA cohort, and 13% for the control cohort. The higher proportion in the control cohort is likely due to the lack of GP or hospital visits from not having a chronic condition. However, when the missing values were excluded, the proportion of different ethnicities between the JIA and the control cohort were similar. In addition, ethnicity was not included in the Cox-proportional hazard model so should not have biased any estimates of mortality. Deaths were acquired through linkage with the national death register for complete capture of deaths otherwise missing from CPRD, and included cause of death. This meant that patients lost to follow-up within CPRD itself could continue to contribute person time up until the cut-off date for the linked death data. However, comparisons of causes of death between cohorts were not considered appropriate due to small numbers. Until 2011, only tumour necrosis factor (TNF) inhibitors were available for the treatment of systemic JIA. However, it has since been shown that these patients may benefit from treatment with interleukin (IL) inhibitors-1 or -6 rather than TNF inhibitors, and since 2015 have been the recommended first biologic of choice for these patients according to the NHS England treatment pathway [15]. In May 2011, the IL-6 inhibitor tocilizumab was approved for patients with systemic JIA [12], and in 2018, after the cut-off date for this analysis, the IL-1 inhibitor anakinra was approved for patients with systemic JIA [16], although patients were using anakinra in routine clinical practice before then [17]. A common side effect of systemic JIA is the occurrence of macrophage activation syndrome (MAS), which occurs approximately in up to a quarter of this patient population [[18], [19], [20]]. However, IL-1 and IL-6 inhibitors have revolutionised MAS treatment, prevention, as well as disease activity outcomes in patients systemic JIA [17]. Additionally, children with refractory disease were historically treated with high-dose glucocorticoid steroids, which have significant adverse effects, or in the most severe cases, would have a stem cell transplant. Both these factors have been highlighted previously as causes of death for children with JIA, for example Davies et al [14] reported 2 deaths from complications post-stem cell transplant and one from MAS. Therefore, better treatment for MAS and reduced use of high-dose steroids are likely to be the reason for a reduced mortality rate observed in more recent years.

This is the first analysis to calculate the all-cause mortality rate for children, young people, and young adults with JIA in England. Overall death in this age group was very rare, although higher in those with systemic JIA. It is possible that the mortality rates in JIA have decreased over the past 20 years; although the reasons for this decrease cannot be deciphered from this analysis, changes to the management of JIA and in particular systemic JIA may have contributed. <END ARTICLE>

## Competing interests

LKF, NS, JL, and MJ report no conflicts of interest. LRW reports honoraria from Pfizer, paid to UCL, unrelated to this manuscript and consultancy fees from Pfizer and Cabaletta, paid to UCL, unrelated to this manuscript. KLH reports honoraria from Abbvie and grants from Pfizer and BMS unrelated to this manuscript, and is supported by the NIHR Manchester Biomedical Research Centre (NIHR203308). JHH reports consulting work for Versus Arthritis.

## Appendix Supplementary materials

Image, application 1

## Footnotes

Supplementary material associated with this article can be found in the online version at doi:10.1016/j.ero.2025.04.001.
