# Supplementary material for: Mortality rates in children, young people, and young adults with JIA: an observational study using the Clinical Practice Research Datalink (CPRD)
Source: EULAR Rheumatol Open. 2025 May 14;1(2):39–44. doi: 10.1016/j.ero.2025.04.001 (PMC13292137; doi:10.1016/j.ero.2025.04.001)
Supplement: Supplementary file 1 [file mmc1.docx]

**Supplementary Materials**

### **Supplementary Table 1: CPRD GOLD Read Codes used to identify potential patients with JIA; including systemic JIA codes.**

| **Read Code “medcode”** | **Description** | **Systemic JIA code** |
| --- | --- | --- |
| 476 | psoriatic arthropathy |  |
| 587 | arthritis |  |
| 844 | rheumatoid arthritis |  |
| 1670 | polyarthritis |  |
| 2184 | ankylosing spondylitis |  |
| 2294 | arthritis of spine |  |
| 2474 | wrist arthritis nos |  |
| 2852 | knee arthritis nos |  |
| 3543 | foot arthritis nos |  |
| 3853 | unspecified monoarthritis |  |
| 4186 | juvenile rheumatoid arthritis - still's disease | Systemic JIA |
| 4578 | sero negative arthritis |  |
| 4652 | chronic arthritis |  |
| 6916 | seronegative rheumatoid arthritis |  |
| 7196 | other juvenile arthritis |  |
| 7334 | hip arthritis nos |  |
| 8969 | hand arthritis nos |  |
| 8990 | shoulder arthritis nos |  |
| 9707 | seropositive errosive rheumatoid arthritis |  |
| 10555 | periarthritis of shoulder |  |
| 10919 | sero negative polyarthritis |  |
| 11257 | ankle arthritis nos |  |
| 11269 | acute arthritis |  |
| 12019 | seropositive rheumatoid arthritis, unspecified |  |
| 12500 | psoriatic arthropathy nos |  |
| 12575 | juvenile arthritis in crohn's disease |  |
| 14900 | periarthritis nos |  |
| 15390 | unspecified monoarthritis of the shoulder region |  |
| 16583 | periarthritis of wrist |  |
| 16591 | elbow arthritis nos |  |
| 17230 | generalised arthritis |  |
| 21358 | rheumatoid arthritis of shoulder |  |
| 21503 | arthritis mutilans |  |
| 21533 | pauciarticular juvenile rheumatoid arthritis |  |
| 23812 | unspecified monoarthritis of the upper arm |  |
| 23834 | adult still's disease | Systemic JIA |
| 25020 | unspecified polyarthropathy or polyarthritis |  |
| 27557 | juvenile rheumatoid arthritis nos |  |
| 27603 | rheumatoid arthritis and other inflammatory polyarthropathy |  |
| 28456 | juvenile arthritis in psoriasis |  |
| 29443 | unspecified monoarthritis nos |  |
| 29480 | unspecified monoarthritis of the hand |  |
| 29647 | unspecified monoarthritis of the ankle and foot |  |
| 29949 | unspecified monoarthritis of the lower leg |  |
| 31054 | rheumatoid arthritis - multiple joint |  |
| 31181 | juvenile seronegative polyarthritis |  |
| 31360 | juvenile rheumatoid arthritis |  |
| 32001 | adult-onset still's disease | Systemic JIA |
| 32149 | distal interphalangeal psoriatic arthropathy |  |
| 35936 | unspecified polyarthropathy or polyarthritis nos |  |
| 36276 | monarticular juvenile rheumatoid arthritis |  |
| 37431 | rheumatoid arthropathy + visceral/systemic involvement nos | Systemic JIA |
| 40301 | unspecified monoarthritis of other specified site |  |
| 41941 | rheumatoid arthritis of pip joint of finger |  |
| 42299 | rheumatoid arthritis of mcp joint |  |
| 42405 | juvenile ankylosing spondylitis |  |
| 43652 | unspecified monoarthritis of the forearm |  |
| 44203 | other rheumatoid arthritis of spine |  |
| 44743 | rheumatoid arthritis of cervical spine |  |
| 46622 | pauciarticular onset juvenile chronic arthritis |  |
| 47831 | acute polyarticular juvenile rheumatoid arthritis |  |
| 48832 | rheumatoid arthritis of wrist |  |
| 49067 | rheumatoid arthritis of hip |  |
| 49227 | other rheumatoid arthropathy + visceral/systemic involvement | Systemic JIA |
| 50644 | juvenile rheumatoid arthropathy unspecified |  |
| 50863 | rheumatoid arthritis of knee |  |
| 51238 | rheumatoid arthritis of 1st mtp joint |  |
| 51239 | rheumatoid arthritis of ankle |  |
| 56202 | [x]seropositive rheumatoid arthritis, unspecified |  |
| 59107 | [x]other psoriatic arthropathies |  |
| 59738 | rheumatoid arthritis of elbow |  |
| 63198 | rheumatoid arthritis of dip joint of finger |  |
| 63365 | rheumatoid arthritis of distal radio-ulnar joint |  |
| 63635 | unspecified monoarthritis of unspecified site |  |
| 64223 | unspecified monoarthritis of the pelvic region and thigh |  |
| 69960 | [x]other specified arthritis |  |
| 70221 | [x]other specified rheumatoid arthritis |  |
| 70658 | rheumatoid arthritis of talonavicular joint |  |
| 71083 | juvenile arthritis in ulcerative colitis |  |
| 71784 | rheumatoid arthritis of other tarsal joint |  |
| 73619 | rheumatoid arthritis of subtalar joint |  |
| 93715 | [x]other seropositive rheumatoid arthritis |  |
| 94334 | suspected inflammatory arthritis |  |
| 94854 | [x]other juvenile arthritis |  |
| 96456 | endemic polyarthritis |  |
| 96880 | psoriatic arthritis |  |
| 99414 | rheumatoid arthritis of lesser mtp joint |  |
| 100776 | rheumatoid arthritis of sacro-iliac joint |  |
| 100914 | rheumatoid arthritis of acromioclavicular joint |  |
| 106440 | [x]rheumatoid arthritis+involvement/other organs or systems |  |
| 107112 | rheumatoid arthritis of ip joint of toe |  |
| 107791 | rheumatoid arthritis of tibio-fibular joint |  |
| 107963 | rheumatoid arthritis of sternoclavicular joint |  |
| 109023 | axial spondyloarthritis |  |

### **Supplementary Table 2: CPRD Aurum Read Codes used to identify potential patients with JIA; including systemic JIA codes.**

| **Read Code “medcodeid”** | **Description** | **Systemic JIA code** |
| --- | --- | --- |
| 1464018 | unspecified monoarthritis |  |
| 7278014 | arthritis |  |
| 16833013 | ankylosing spondylitis |  |
| 20587019 | acute arthritis |  |
| 32869011 | arthritis mutilans |  |
| 55628018 | psoriatic arthritis |  |
| 59922014 | chronic arthritis |  |
| 116082011 | rheumatoid arthritis |  |
| 123542016 | pauciarticular juvenile rheumatoid arthritis |  |
| 125937016 | acute polyarticular juvenile rheumatoid arthritis |  |
| 178460018 | periarthritis of wrist |  |
| 309787016 | rheumatoid arthritis of cervical spine |  |
| 309788014 | other rheumatoid arthritis of spine |  |
| 309789018 | rheumatoid arthritis of shoulder |  |
| 309790010 | rheumatoid arthritis of sternoclavicular joint |  |
| 309791014 | rheumatoid arthritis of acromioclavicular joint |  |
| 309792019 | rheumatoid arthritis of elbow |  |
| 309794018 | rheumatoid arthritis of wrist |  |
| 309798015 | rheumatoid arthritis of hip |  |
| 309800010 | rheumatoid arthritis of knee |  |
| 309802019 | rheumatoid arthritis of ankle |  |
| 309803012 | rheumatoid arthritis of subtalar joint |  |
| 309804018 | rheumatoid arthritis of talonavicular joint |  |
| 309805017 | rheumatoid arthritis of other tarsal joint |  |
| 309824019 | juvenile rheumatoid arthropathy unspecified |  |
| 309827014 | monarticular juvenile rheumatoid arthritis |  |
| 309828016 | juvenile rheumatoid arthritis nos |  |
| 309829012 | other juvenile arthritis |  |
| 309833017 | juvenile arthritis in crohn's disease |  |
| 309836013 | juvenile arthritis in ulcerative colitis |  |
| 310159018 | generalised arthritis |  |
| 310166017 | unspecified monoarthritis of unspecified site |  |
| 310167014 | unspecified monoarthritis of the shoulder region |  |
| 310168016 | unspecified monoarthritis of the upper arm |  |
| 310169012 | unspecified monoarthritis of the forearm |  |
| 310170013 | unspecified monoarthritis of the hand |  |
| 310171012 | unspecified monoarthritis of the pelvic region and thigh |  |
| 310172017 | unspecified monoarthritis of the lower leg |  |
| 310173010 | unspecified monoarthritis of the ankle and foot |  |
| 310174016 | unspecified monoarthritis of other specified site |  |
| 310175015 | unspecified monoarthritis nos |  |
| 311398011 | periarthritis nos |  |
| 312518013 | [x]other seropositive rheumatoid arthritis |  |
| 312520011 | [x]other specified rheumatoid arthritis |  |
| 312523013 | [x]other juvenile arthritis |  |
| 312529012 | [x]other specified arthritis |  |
| 312534011 | [x]seropositive rheumatoid arthritis, unspecified |  |
| 359167017 | foot arthritis nos |  |
| 359168010 | ankle arthritis nos |  |
| 359169019 | knee arthritis nos |  |
| 359170018 | hip arthritis nos |  |
| 359171019 | wrist arthritis nos |  |
| 359172014 | elbow arthritis nos |  |
| 359292012 | seronegative rheumatoid arthritis |  |
| 359310019 | juvenile arthritis in psoriasis |  |
| 359312010 | juvenile ankylosing spondylitis |  |
| 378099017 | endemic polyarthritis |  |
| 400192018 | unspecified polyarthropathy or polyarthritis nos |  |
| 405142017 | unspecified polyarthropathy or polyarthritis |  |
| 426510015 | rheumatoid arthritis - multiple joint |  |
| 451461014 | seropositive errosive rheumatoid arthritis |  |
| 502690012 | pauciarticular onset juvenile chronic arthritis |  |
| 1209790019 | arthritis of spine |  |
| 1786700015 | periarthritis of shoulder |  |
| 2472447010 | juvenile rheumatoid arthritis |  |
| 2472449013 | juvenile seronegative polyarthritis |  |
| 2549731014 | undifferentiated inflammatory arthritis |  |
| 16911000006114 | other rheumatoid arthropathy + visceral/systemic involvement | Systemic JIA |
| 140551000006117 | shoulder arthritis nos |  |
| 149911000006117 | seropositive rheumatoid arthritis, unspecified |  |
| 162041000006117 | rheumatoid arthritis of dip joint of finger |  |
| 162051000006115 | rheumatoid arthritis of distal radio-ulnar joint |  |
| 162081000006111 | rheumatoid arthritis of ip joint of toe |  |
| 162101000006115 | rheumatoid arthritis of lesser mtp joint |  |
| 162111000006117 | rheumatoid arthritis of mcp joint |  |
| 162131000006111 | rheumatoid arthritis of pip joint of finger |  |
| 162141000006118 | rheumatoid arthritis of sacro-iliac joint |  |
| 162191000006110 | rheumatoid arthritis of tibio-fibular joint |  |
| 162231000006117 | rheumatoid arthropathy + visceral/systemic involvement nos | Systemic JIA |
| 168751000006115 | rheumatoid arthritis and other inflammatory polyarthropathy |  |
| 168761000006118 | rheumatoid arthritis of 1st mtp joint |  |
| 215551000000114 | sero negative polyarthritis |  |
| 219271000000117 | sero negative arthritis |  |
| 221661000006114 | polyarthritis |  |
| 424981000006114 | [x]rheumatoid arthritis+involvement/other organs or systems |  |
| 463281000006113 | adult still's disease | Systemic JIA |
| 463301000006112 | adult-onset still's disease | Systemic JIA |
| 559621000000119 | suspected inflammatory arthritis |  |
| 755441000006119 | juvenile rheumatoid arthritis - still's disease | Systemic JIA |
| 815501000006110 | hand arthritis nos |  |
| 856221000006115 | sero-negative polyarthritis |  |
| 889621000006117 | arthritis/arthrosis |  |
| 889731000006111 | rheumatoid arthritis nos |  |
| 889841000006113 | still's disease - juvenile r.a | Systemic JIA |
| 905291000006113 | [rfc] arthritis |  |
| 905301000006114 | [rfc] rheumatoid |  |
| 909191000006119 | [rfc] rheumatoid arthritis |  |
| 909461000006119 | [rfc] arthritis |  |
| 1898101000006110 | axial spondyloarthritis |  |
| 1909381000006115 | non-radiographic axial spondyloarthritis |  |
| 2360741000000115 | axial spondyloarthritis |  |

### **Supplementary Table 3: CPRD GOLD Read Codes used to identify potential patients with asthma.**

| **Read Code “medcode”** | **Description** |
| --- | --- |
| 78 | asthma |
| 1208 | Childhood asthma |
| 1555 | bronchial asthma |
| 2290 | allergic asthma |
| 3018 | mild asthma |
| 3366 | severe asthma |
| 3458 | occasional asthma |
| 3665 | late onset asthma |
| 4442 | asthma unspecified |
| 4606 | exercise induced asthma |
| 5267 | intrinsic asthma |
| 5627 | hay fever with asthma |
| 5798 | chronic asthmatic bronchitis |
| 5867 | exercise induced asthma |
| 6707 | extrinsic asthma with asthma attack |
| 7146 | extrinsic (atopic) asthma |
| 7731 | pollen asthma |
| 11370 | asthma confirmed |
| 12987 | late-onset asthma |
| 13065 | moderate asthma |
| 14777 | extrinsic asthma without status asthmaticus |
| 15248 | hay fever with asthma |
| 16070 | asthma nos |
| 18323 | intrinsic asthma with asthma attack |
| 21232 | allergic asthma nec |
| 22752 | occupational asthma |
| 27926 | extrinsic asthma with status asthmaticus |
| 29325 | intrinsic asthma without status asthmaticus |
| 39478 | wood asthma |
| 40823 | brittle asthma |
| 41017 | aspirin induced asthma |
| 45073 | intrinsic asthma nos |
| 45782 | extrinsic asthma nos |
| 47684 | detergent asthma |
| 58196 | intrinsic asthma with status asthmaticus |
| 73522 | work aggravated asthma |
| 93353 | sequoiosis (red-cedar asthma) |

**Supplementary Table 4: CPRD Aurum Read Codes used to identify potential patients with asthma.**

| **Read Code “medcodeid”** | **Description** |
| --- | --- |
| 21390015 | Intrinsic asthma without status asthmaticus |
| 69311016 | Detergent asthma |
| 94731013 | Wood asthma |
| 95786019 | Occupational asthma |
| 98546013 | Intrinsic asthma with status asthmaticus |
| 104872017 | Extrinsic asthma without status asthmaticus |
| 151338014 | Extrinsic asthma with status asthmaticus |
| 264565016 | Emergency asthma admission since last appointment |
| 283550015 | Emergency admission, asthma |
| 285239019 | Seen in asthma clinic |
| 285727014 | Attends asthma monitoring |
| 285891014 | Patient in asthma study |
| 301450011 | Chronic asthmatic bronchitis |
| 301480018 | Bronchial asthma |
| 301499010 | Extrinsic asthma NOS |
| 301508013 | Intrinsic asthma NOS |
| 301509017 | Mixed asthma |
| 338238011 | Brittle asthma |
| 350147016 | Childhood asthma |
| 350148014 | Late onset asthma |
| 350149018 | Late-onset asthma |
| 350151019 | Extrinsic asthma with asthma attack |
| 350152014 | Allergic asthma NEC |
| 350153016 | Hay fever with asthma |
| 350154010 | Pollen asthma |
| 350156012 | Intrinsic asthma with asthma attack |
| 396114013 | Intrinsic asthma |
| 396118011 | Status asthmaticus NOS |
| 419211018 | Acute exacerbation of asthma |
| 1208962015 | Number of asthma exacerbations in past year |
| 1208969012 | Mild asthma |
| 1208970013 | Moderate asthma |
| 1208971012 | Occasional asthma |
| 1208972017 | Severe asthma |
| 1483199016 | Allergic asthma |
| 1484905010 | Change in asthma management plan |
| 1484910014 | Step up change in asthma management plan |
| 1484911013 | Step down change in asthma management plan |
| 1484953014 | Absent from work or school due to asthma |
| 2533402016 | Referral to asthma clinic |
| 145961000006117 | Severe asthma attack |
| 149741000006116 | Sequoiosis (red-cedar asthma) |
| 235661000000118 | Does not have asthma management plan |
| 536221000000110 | Royal College of Physicians asthma assessment |
| 655601000006113 | Exercise induced asthma |
| 655611000006111 | Exercise induced asthma |
| 660351000006118 | Extrinsic (atopic) asthma |
| 797211000006117 | Further asthma - drug prevent. |
| 817361000006114 | Hay fever with asthma |
| 885291000006115 | Extrinsic asthma - atopy |
| 929091000006111 | Aspirin induced asthma |
| 955631000006116 | Occupational asthma |
| 983771000006118 | Work aggravated asthma |
| 1139051000000110 | Mini asthma quality of life questionnaire |
| 1139131000000110 | Under care of asthma specialist nurse |
| 1176461000000110 | Patient has a written asthma personal action plan |
| 1766051000006110 | Royal College Physician asthma assessment 3 question score |
| 1769451000000110 | Seen in school asthma clinic |
| 1807891000006110 | Asthma causes daytime asthma symptoms less than weekly |
| 1807911000006110 | Asthma causes daytime asthma symptoms daily |
| 1807941000006110 | Asthma causes night time asthma symptoms weekly or more often |
| 1811901000006110 | Number days absent from school due to asthma in past 6 month |
| 1821501000006110 | Frequent night time asthma symptoms |
| 1821511000006110 | Infrequent asthma exacerbations |
| 1821521000006110 | Occasional asthma exacerbations |
| 1821531000006110 | Frequent asthma exacerbations |
| 1856351000006110 | Follow-up asthma assessment |
| 1859261000006110 | Date of asthma diagnosis |
| 1880001000006110 | No change in asthma management plan |
| 1916511000006110 | Access to online patient asthma education given |
| 1955191000006110 | Keele ENHANCE trial - asthma review |
| 2004171000006110 | Advance supply of asthma medication |
| 2009981000006110 | Difficult asthma |
| 2010031000006110 | Acute infective exacerbation of asthma |
| 2010041000006110 | Acute non-infective exacerbation of asthma |
| 2011441000006110 | OH respiratory questionnaire: history of asthma |
| 2240591000000110 | Chronic asthma with fixed airflow obstruction |
| 2423691000000110 | Telehealth asthma monitoring |
| 2460311000000110 | At risk of severe asthma exacerbation |
| 2460351000000110 | Severe asthma exacerbation risk assessment |
